# Dental caries experience and socio-economic status among Iranian children: a multilevel analysis

**DOI:** 10.1186/s12889-019-7693-1

**Published:** 2019-11-27

**Authors:** Majid Ghasemianpour, Soheila Bakhshandeh, Armin Shirvani, Naghmeh Emadi, Hamid Samadzadeh, Nadereh Moosavi Fatemi, Anoosheh Ghasemian

**Affiliations:** 1 Dental Research Center, Research Institute of Dental Sciences, Shahid Beheshti University of Medical Sciences, Tehran, Iran; 2 Dentofacial Deformities Research Center, Research Institute of Dental Sciences, Dental School, Shahid Beheshti University of Medical Sciences, Tehran, Iran; 3 Iranian Center for Endodontic Research, Research Institute of Dental Sciences, Faculty of Medical Education, Shahid Beheshti University of Medical Sciences, Tehran, Iran; 4 Preventive Dentistry Research Center, Research Institute of Dental Sciences, Dental School, Shahid Beheshti University of Medical Sciences, Tehran, Iran

**Keywords:** Dental caries, Socio-economic factors, Multilevel analysis

## Abstract

**Background:**

Socio-economic factors are considered as main determinants causing disparities in oral health across different countries. The aim of the present study was to investigate the associations of social and economic factors with dental caries experience among 6- and 12-year-old Iranian children.

**Methods:**

In this cross-sectional study, a total of 31,146 students, aged 6 and 12 years old, were enrolled from all (31) provinces in Iran. Based on the standardized World Health Organization (WHO) criteria for oral health surveys, dental caries indices in primary and permanent teeth were assessed by clinical examination in schools. Data on socio-economic status was obtained from the modified WHO questionnaire and national data bank. The decayed, missing and filled teeth (dmft/DMFT) indices for primary and permanent dentition were compared at the individual and provincial levels using multilevel regression analysis. Poisson regression analysis was used to evaluate the association of social (demographic and behavioral) determinants with dental caries indices among individuals. To assess the causes of difference in dental caries indices across provinces, justifiable economic factors were also analyzed using poisson regression analysis.

**Results:**

The mean (SE) of dmft and DMFT were 5.84 (0.05) and 1.84 (0.03), for 6-and 12-year-old children, respectively. The differences of dental caries indices were statistically significant among provinces. Higher level of parental education was negatively related to dental caries indices of both age groups. Rural residency was positively and dental flossing was reversely associated with dmft index of 6-year-old children. Negative associations were found between frequency of tooth brushing and preventive dental utilization with dmft and DMFT indices. Gross Domestic Product (GDP) index had negative and Consumer Price Index (CPI) had positive associations with dmft and DMFT indices in both age groups. However, positive relationships were observed between Gini index with DMFT index among 12-year-old children; as well as between the number of dentists per capita with dmft index among 6-year-old children.

**Conclusion:**

Socio-demographic and behavioral factors were found to be associated with dental caries experience. However, economic indicators had the greatest importance.

## Background

From the public health perspective, oral diseases are one of the most prevalent diseases worldwide, with extensive social and economic effects. However, policies related to oral disease prevention have been mostly overlooked by public health policy makers [1]. According to the Global Burden of Disease (GBD) study 2010, untreated dental caries in permanent teeth is the most common disease in all human societies [2]. In addition, dental caries prevalence, resulted from oral health surveys, is essential to evaluate oral health status of populations. Assessment of such prevalence is required to evaluate its determinants, design/formulate effective preventive programmes and predict possible future needs for oral health care [3]. Furthermore, based on a report by World Health Organization (WHO), dental treatments have always been the most expensive treatments among all chronic diseases [4], and impose great economic burden on both individuals and health care systems [5].

In Iran, and during the past two decades, particularly after the implementation of policy for the rapid increase in the number of dental schools, a large number of dentists have been trained without any significant influence on reduction of dental caries. Moreover, and during the mentioned period, programs based on prevention of oral diseases and evaluation of effective determinants on oral diseases have not been taken into account. In a study conducted based on the GBD study 2010, the rate of dental caries increased in Iran from 1990 to 2010 [6].

Socio-economic determinants have always been referred to substantial factors for causing inequalities in oral health status across different countries [7]. However, controversial results from their impacts have been reported in various studies. A systematic review, on the evaluation of oral health status of 83 developed countries and 66 developing countries in 2015, indicated that low socio-economic status (SES) was significantly associated with high risk of dental caries, which was more prominent in developed countries [8]. Conversely, in another study conducted in 2014, which evaluated oral health status of children and adolescents aged 6 to 17, dental caries was more prevalent among children with moderate and high SES compared to similar group of low SES. The possible reason was attributed to more consumption of sugar-based starchy food among the upper social class individuals [9].

The aim of the present study was to investigate the dental caries status using decayed, missing and filled teeth (dmft/DMFT) indices for 6- and 12-year-old Iranian children across all provinces nationwide, and assess the impact of social (demographic, behavioral) and economic determinants on dental caries experience, based on the results from the National Oral Health survey 2016 in accordance with the WHO proposed method. It is expected that the findings of this study would be useful for implementation of national and regional policies by dental health care providers.

## Methods

### Study design and population

This cross-sectional study was carried out in Iran, using a representative sample of 6- and 12-year-old school children nationwide. The sampling method was stratified-cluster random; in which 31 provinces were considered as strata and their districts as clusters. For sample size calculation, 95% confidence interval and absolute error of 5%, and standard deviation of dmft equal to 0.3 (according to a previous survey) was adapted. In addition, due to about 88% response rate and desisgn effect of 1.5, approximately 250 subjects were needed in each age and sex group. As we had 4 age and sex categories, the final sample size was calculated to be 1000 (except Tehran province, recruited 1500 samples).

Blocks were defined as the primary sampling unit; each block consisted of five clusters. The clusters were considered as the secondary sampling unit. In each province, 10 blocks were distributed among districts by systematic random method proportional to the size of population_so that districts with more population had more blocks. Districts were categorized in order to choose the ones with different demographic and SES levels. The clusters chosen based on the number of defined blocks in each district, both in urban and rural areas. Each cluster composed of a boys’ and a girls’ primary school for random sampling of 5 students in both age groups.

In this study, schools were considered as sample listing units, and their lists were available to each province. Thus, 50 boys’ schools and 50 girls’ schools were chosen from each province. For selection of clusters in each district, one school was initially chosen as cluster-head using systematic method proportional to the size among cities and villages in that district, and the nearest school was consequently selected. A list of primary schools was used for sampling of proportional schools in each province. Similarly, cities and villages with different SES had the same chance to be chosen. Students were also randomly selected inside the schools for participation in this study. Four dentists, with Cohen’s kappa coefficient (κ) of 87.6%, trained and calibrated the examiners through 5 educational workshops. The Cohen’s kappa statistic (κ) for inter-rater reliability of 207 examiners with dentists was between 0.65 and 0.83 in all workshops.

### Data collection

Dental caries indices for primary and permanent dentition (dmft/DMFT) were measured by clinical examinations inside the schools. The examinations were based on the standardized international criteria proposed by WHO for oral health surveys using WHO probe, disposable mouth mirrors and gauze pads [3]. The modified WHO questionnaire and national data bank were used for collection of demographic, behavioral and economic information. Sex, fathers’ education, mothers’ education, place of residence (urban/rural), oral hygiene behaviors (use of tooth brush, frequency of toothbrushing, and use of dental floss), and use of preventive services (fissure sealant and fluoride varnish therapy) were used as social (demographic and behavioral) determinants. Education level of fathers and mothers were categorized into seven groups of illiterate, reading and writing literacy, elementary school, secondary school, diploma, bachelor degree and master degree and more. Use of tooth brush, dental floss and preventive services were considered as two groups of Yes or No. Frequency of tooth brushing was categorized into two groups of more than once a day or less.

Gini Index was the first economic factor used in this study. This is an indicator of wealth distribution among populations and a measure of income inequality at the provincial level (urban vs rural areas). The high rate of Gini index represents a great social disparity and severe income inequality in a country. Gross Domestic Product (GDP) index was the next economic indicator, which is of particular importance among macroeconomic indicators. It determines the value of all finalized goods and services produced within borders of a country at a specific period of time, depending on the currency of that country. This index is not only used as one of the essential indicators of financial performance of a country in various analyses and evaluations, but also is considered along with many other macroeconomic items as its adjacent products.

The third economic indicator was the price index for consumer goods and services; simply called Consumer Price Index (CPI), which is a scale for inflation rate and purchasing power of domestic currency in public and health sectors of any country. It merely evaluates price changes of consumer goods and service packages in health sector. Given the importance of human resources required for provision of health services and the significant impact of manpower on prevention of diseases, the number of dentists per capita in each country was considered the final economic factor. The number of dentists per 10,000 population is presented by the dentist per capita index. All economic factors were considered as continuous variables.

It should be mentioned that all demographic and economic data used in the present study were extracted from the Iranian Statistical Census Database in 2016, published by the Statistical Center of Iran (https://www.amar.org.ir/english/Iran-Statistical-Yearbook).

## Statistical analysis

Calculation of national mean dmft/DMFT followed the principles of weighing samples in surveys. To assess the relationships between underlying variables of dental caries along with geographical distribution (geographical justification) and dmft/DMFT indices, multilevel regression analysis was applied at the individual and provincial levels. Since dental caries indices are count data and have poisson distribution, the poisson regression analysis was conducted. Mixed effect poisson regression analysis was used to evaluate the impacts of demographic and behavioral factors with dmft/DMFT, at the individual level. Poisson regression analysis was also performed to investigate the effects of justifiable economic factors of geographical distribution; including Gini index (by urban and rural areas), GDP index, CPI index (by urban and rural areas) and dentist per capita index, with dmft/DMFT, at the provincial level.

In the crude model, we only considered the geographical distribution of the dependent variables, dmft/DMFT. In the multivariate model, demographic and behavioral variables including sex, fathers’ education, mothers’ education, place of residence, oral hygiene habits and use of preventive services entered the model. Results for regression models were presented as β-coefficient (β) and standard error (SE). A 2-sided *P*-value < 0.05 was considered the level of statistical significant. All statistical analyses were performed by STATA version 12 software.

## Results

A total of 31,146 Iranian students from all (31) provinces were participated in this study, of which 15,630 school children were 6 years old and 15,508 were 12 years old. Table 1 shows the detailed distributions of mean dmft/DMFT, and percent of each of their components in both age groups of Iranian children by province. The national mean (SE) of dmft and DMFT in 6- and 12-year-old children were 5.84 (0.05) and 1.84 (0.03), respectively. The mean DMFT in 12 provinces were higher than that of the national mean.

**Table 1:** Mean decayed, missing and filled teeth (dmft/DMFT) and percent of their components among 6- and 12-year-old Iranian children by province in the National Oral Health survey 2016

Province	Age							
	6 years				12 years			
	dmft (SE)	%d. dmft (SE)	%m. dmft (SE)	%f. dmft (SE)	DMFT (SE)	%D.DMFT (SE)	%M.DMF (SE)	%F.DMFT (SE)
Azerbaijan, East	5.75 (0.17)	87.93 (1.20)	4.11 (0.61)	7.96 (1.05)	2.18 (0.11)	71.28 (2.16)	4.31 (0.85)	24.41 (2.11)
Azerbaijan, West	7.10 (0.25)	91.34 (0.76)	5.90 (0.59)	2.76 (0.53)	3.31 (0.18)	86.95 (1.46)	3.62 (0.69)	9.43 (1.33)
Ardabil	7.14 (0.24)	91.48 (0.83)	7.12 (0.70)	1.40 (0.44)	2.24 (0.12)	87.55 (1.44)	5.82 (0.95)	6.63 (1.14)
Isfahan	6.01 (0.22)	71.71 (1.54)	8.98 (0.73)	19.32 (1.40)	1.41 (0.12)	61.75 (2.58)	1.21 (0.38)	37.03 (2.57)
Alborz	4.80 (0.23)	81.89 (1.51)	2.37 (0.46)	15.74 (1.44)	1.36 (0.11)	76.48 (2.48)	1.13 (0.63)	22.40 (2.45)
Ilam	6.73 (0.23)	88.74 (0.86)	9.00 (0.75)	2.26 (0.45)	2.24 (0.16)	87.00 (1.55)	2.44 (0.51)	10.56 (1.48)
Bushehr	4.83 (0.19	90.19 (1.12)	4.02 (0.65)	5.79 (0.93)	1.31 (0.1)	87.93 (1.83)	0.07 (0.07)	12.00 (1.82)
Tehran	4.71 (0.2)	67.86 (1.48)	6.40 (0.64)	25.73 (1.44)	1.56 0.09)	61.67 (2.24)	1.54 (0.47)	36.78 (2.25)
Chahar Mahaal and Bakhtiari	6.90 (0.21)	84.68 (1.15)	8.20 (0.74)	7.12 (0.88)	2.19 (0.11)	76.59 (1.89)	4.72 (0.71)	18.69 (1.79)
Khorasan, South	5.80 (0.18)	88.16 (1.09)	5.93 (0.64)	5.91 (0.89)	1.27 (0.08)	70.39 (2.40)	6.65 (1.19)	22.96 (2.28)
Khorasan, Razavi	5.74 (0.18)	86.74 (1.10)	6.81 (0.72)	6.45 (0.89)	1.71 (0.11)	77.28 (2.11)	6.03 (1.17)	16.69 (1.90)
Khorasan, North	4.72 (0.22)	87.53 (1.09)	8.51 (0.81)	3.96 (0.77)	1.61 (0.1)	77.83 (2.16)	5.39 (1.03)	16.78 (2.03)
Khuzestan	5.49 (0.23)	89.94 (1.01)	5.89 (0.69)	4.17 (0.74)	2.21 (0.17)	93.45 (1.11)	3.02 (0.69)	3.54 (0.88)
Zanjan	5.85 (0.22)	87.62 (1.13)	6.08 (0.65)	6.30 (0.92)	1.56 (0.12)	76.72 (2.23)	4.06 (0.89)	19.22 (2.16)
Semnan	6.00 (0.26)	85.38 (1.20)	6.39 (0.70)	8.23 (0.99)	1.78 (0.12)	81.88 (2.00)	3.01 (0.73)	15.12 (1.91)
Sistan and Baluchestan	5.50 (0.24)	93.65 (0.82)	4.54 (0.66)	1.81 (0.49)	1.46 (0.1)	93.97 (1.31)	1.32 (0.50)	4.72 (1.16)
Fars	5.63 (0.25)	83.66 (1.26)	8.74 (0.88)	7.60 (0.98)	1.62 (0.13)	78.79 (2.36)	1.96 (0.56)	19.25 (2.31)
Qazvin	4.98 (0.24)	86.99 (1.18)	7.26 (0.76)	5.75 (0.90)	1.81 (0.11)	84.29 (1.77)	2.53 (0.70)	13.18 (1.69)
Qom	6.05 (0.23)	80.34 (1.35)	7.59 (0.72)	12.07 (1.19)	2.13 (0.14)	80.81 (2.01)	2.63 (0.66)	16.55 (1.95)
Kurdistan	7.41 (0.31)	92.71 (0.69)	5.17 (0.53)	2.12 (0.43)	3.39 (0.2)	87.29 (1.33)	3.03 (0.62)	9.68 (1.23)
Kerman	6.26 (0.2)	90.55 (0.92)	6.88 (0.75)	2.57 (0.55)	1.49 (0.11)	86.30 (1.84)	3.67 (0.91)	10.03 (1.69)
Kermanshah	5.64 (0.24)	88.80 (1.08)	6.04 (0.65)	5.16 (0.86)	1.82 (0.16)	83.38 (1.80)	5.08 (1.06)	11.54 (1.57)
Kohgiluyeh and BoyerAhmad	6.21 (0.35)	89.38 (1.01)	7.07 (0.75)	3.55 (0.74)	2.00 (0.17)	84.47 (1.91)	2.48 (0.66)	13.05 (1.83)
Golestan	7.33 (0.23)	92.25 (0.87)	3.93 (0.54)	3.83 (0.67)	2.09 (0.11)	88.56 (1.56)	2.82 (0.78)	8.62 (1.41)
Gilan	5.42 (0.17)	84.20 (1.15)	7.82 (0.75)	7.97 (0.93)	1.38 (0.11)	80.87 (2.14)	3.22 (0.86)	15.91 (2.01)
Lorestan	5.72 (0.22)	91.26 (0.77)	7.21 (0.67)	1.53 (0.41)	1.33 (0.10)	84.03 (1.93)	5.31 (1.05)	10.66 (1.68)
Mazandaran	7.19 (0.26)	86.88 (1.01)	7.38 (0.65)	5.74 (0.77)	2.96 (0.18)	87.49 (1.38)	2.76 (0.58)	9.75 (1.26)
Markazi	5.22 (0.22)	87.17 (1.18)	4.84 (0.61)	7.99 (1.0)	1.24 (0.09)	72.47 (2.64)	2.46 (0.84)	25.07 (2.57)
Hormozgān	5.61 (0.22)	90.51 (1.06)	3.51 (0.46)	5.98 (0.91)	0.94 (0.11)	92.60 (1.63)	1.53 (0.68)	5.87 (1.49)
Hamadan	6.70 (0.26)	85.53 (1.07)	7.99 (0.73)	6.47 (0.87)	1.89 (0.13)	75.37 (2.21)	2.94 (0.71)	21.69 (2.15)
Yazd	6.63 (0.23)	83.32 (1.13)	7.27 (0.63)	9.40 (0.98)	1.66 (0.13)	63.83 (2.48)	4.18 (0.97)	31.98 (2.46)
Total	5.84 (0.05)	85.14 (0.31)	6.37 (0.18)	8.49 (0.28)	1.84 (0.03)	79.57 (0.54)	3.11 (0.19)	17.33 (0.51)

Of the participants, 90.9% of 6-year-old children and 92.1% of 12-year-old children cleaned their teeth with toothbrush, while only 11% of 6-year-old children and 18.6% of 12-year-old children used dental floss. Tooth brushing more than once a day was reported 47.9 and 55.2% among 6- and 12-year-old students, respectively. In addition, utilization of preventive dental services was 16.6% in 6-year-old children and 11% in children who were 12 years old.

Both crude and adjusted multilevel regression analyses were performed to evaluate differences of dmft/DMFT at the provincial and individual levels. In the crude model, only geographical distribution indicators were considered, while the multivariate model were adjusted for all individual variables, in addition to considering geographical distribution indicators. The results indicated that in both models, differences of dmft and DMFT were statistically significant across provinces of the country and among individuals of the provinces (*P* < 0.001).

Table 2 demonstrates an overview of the associations between demographic, behavioral and economic factors with dmft/DMFT indices by estimated β and SE for both age groups of children. The poisson regression analysis showed that tooth brushing more than once a day, use of preventive care, and increased GDP index were negatively and significantly associated with dmft and DMFT scores. Conversely, higher CPI had significant positive associations with dmft and DMFT scores.

**Table 2:** β-coefficient (β) and standard error (SE) for the associations of socio-demographic and behavioral factors, as well as justifiable economic factors with mean decayed, missing and filled teeth (dmft/DMFT) among 6-and 12-year-old Iranian children in the National Oral Health survey 2016

	Age			
	6 years old		12 years old	
	dmft		DMFT	
	β (SE)	*P*- value	β (SE)	*P*-value
**Individual level**				
Female	−0.045 (0.008)	< 0.001	0.141 (0.014)	< 0.001
Increased fathers’ Education level	− 0.022 (0.003)	< 0.001	0.003 (0.004)	0.528
Increased mothers’ Education level	0.006 (0.003)	0.058	−0.043 (0.005)	< 0.001
Rural	0.024 (0.009)	0.008	0.026 (0.016)	0.109
Toothbrush	0.007 (0.016)	0.656	−0.091 (0.026)	0.001
Dental floss	−0.052 (0.012)	< 0.001	0.029 (0.017)	0.084
More than once a day Tooth brushing	−0.078 (0.008)	< 0.001	−0.038 (0.014)	0.008
Preventive care	−0.083 (0.010)	< 0.001	−0.206 (0.022)	< 0.001
**Provincial level**				
Increased Gini coefficient	0.162 (0.100)	0.106	0.589 (0.180)	0.001
Number of dentist	0.271 (0.086)	0.002	−0.130 (0.150)	0.387
Increased Log GDP	−0.176 (0.011)	< 0.001	−0.283 (0.019)	< 0.001
Increased consumer price index (CPI)	0.001 (0.000)	< 0.001	0.004 (0.000)	< 0.001
_cons	3.510 (0.142)	< 0.001	2.603 (0.252)	< 0.001

## Discussion

In the present national survey, based on the WHO classification, dental caries indices were categorized into “severe range” for 6-year-old and “low range” for 12-year-old children [10]. The major component of indicators was decayed teeth in both age groups, which were estimated 85.1 and 79.6% in 6- and 12-year-old groups, respectively.

Positive association was observed between DMFT index and 12-year-old female children. The possible explanations for higher mean DMFT among girls could be attributed to relatively earlier tooth eruption, different dietary habits in comparison with boys [11], as well as potential difference in biochemical composition and flow rate of their saliva [12]. Additionally, in a study by Lukacs and Largaespada [11], hormonal fluctuations in girls have been considered as a critical factor for their oral and dental health status. We also found reverse associations of higher level of fathers’ and mothers’ education with dental caries experience of 6-and 12-year-old children, respectively, which is consistent with the results of a systematic review of 25 studies conducted in Iran from 1994 to 2017 [13]. The relationships of education status in Japanese mothers [14] and Greek fathers [15] with dental caries experience also confirmed the role of parents’ and family literacy in oral health promotion.

We also observed that rural residency was positively associated with dmft index, which is similar to the results of a study performed on Polish preschool children [16]. However, DMFT index was not related to place of residence. In contrary to our finding, an exploratory cross-sectional study showed that lower prevalence and severity of dental caries were seen in urban children aged 12 years old compared to rural children with similar age group. The potential reasons were related to lower oral health knowledge and inappropriate behavioral beliefs and practices among rural children [17].

Using dental floss was negatively related to dental caries experience in children aged 6 years old, which is in contrast to the studies of a systematic review in 2017 on caries status in primary teeth in which only one study showed reverse association of dental flossing with dental caries [18]. For children aged 12 years old, no association was observed between their dental caries experience with using dental floss, which is supported by the findings of two Cochrane systematic reviews [19, 20]. However, the role of dental floss was defended by Vernon and Seacat (2017) [21], which evaluated a lack of sufficient evidence on the impact of dental flossing on dental caries reduction. Their research emphasized that none of the studies in the Cochrane systematic reviews was considered dental caries as an outcome variable. Furthermore, no measurement principle was offered for the ability to do proper dental flossing and the information was only based on self-reported approach. Additionally, there was a lack of required guidelines on how to interpret and apply evidence-based findings to specific clinical scenarios in these studies. Therefore, further studies would be essential for evaluating the effect of dental flossing on dental caries.

In the current study, reverse associations were found between higher frequency of tooth brushing with dental caries indices in both age groups, which is supported by the results of a meta-analysis in 2016 that revealed higher frequency of brushing teeth was associated with lower dental caries in both primary and permanent teeth [22]. Similar to tooth brushing, utilization of preventive services was negatively associated with dental caries indices in both groups of children, which is confirmed by the findings of other epidemiological studies [23, 24]. The use of fluoride varnish for prevention of dental caries in primary and permanent dentition is proven by clinical guidelines globally [25], while it’s widespread use at community level is still very slow due to insufficient number of trained personnel. Given the cost-effectiveness of using prophylactic services such as fluoride varnish therapy, especially in high risk populations [26], as well as the importance of eliminating inequality for access to dental care services, oral health system managers and policy makers should allocate resources and reduce barriers to do so.

Considering justifiable economic factors, many epidemiological studies have evaluated the relationships between economic indicators and dental caries or other dental diseases with controversial findings [27–33]. We found that Gini index was positively associated with DMFT index. An ecological study which investigated the relationship between gross national income and distribution of inequalities in society with dental caries, indicated reverse associations between income inequality with DMFT index [27], which is inconsistent with our result. In another study by Vettore and colleagues [28], conducted over 10 years on Brazilian children, Gini index was negatively associated with dental trauma. They suggested that implementation of public health policies for decreasing social inequality could be an influential factor for oral health promotion [28].

In the present study, reverse associations were found between GDP index with dmft and DMFT indices. A study conducted in 2016, for evaluating the effects of social environment and individual factors related to dental pain and dental caries, showed that GDP index was considered as a strong influential determinant [29]. In a study by Masood et al., [30] on investigating the association between per capita consumption of sugar with dental caries, gross national income was much more significant factor than index of inequalities. This finding is supported by the results of the present study, which revealed that as GDP index had significant relationships in most groups in comparison with Gini index, we could confirm its greater impact.

CPI was positively related to dmft and DMFT indices in the current study. Such associations reflected the importance and impact of treatment costs on oral health indices, which is a widespread problem not only in Iran, but also around the world. The global economic impact of oral diseases was estimated 442 billion dollars in 2010 [31]. Based on this report, it is expected that annually, indirect costs _untreated caries in primary and permanent teeth, severe periodontitis, and severe tooth loss_ could make economic loss of additional 144 billion dollars, which is equivalent to the cost of 10 most common causes of death worldwide [31]. As Disability Adjusted Life Year (DALY) is increasing, such numbers are also expected to rise [32].

Positive association was also found between the number of dentists per capita with dmft index. As the most percent of total dmft index is attributed to decayed teeth (85%), this result showed that despite the increasing number of dentists across the country, oral health care services are not desirably available to 6-year-old children. The aforementioned issue is less problematic in 12-year-old age group, as there was no association between the number of dentists per capita with DMFT index. A study which assessed the effects of primary care providers (PCPs), dentists, and both on treatment-related caries among 3- and 5-years-old children, revealed that the rate of treatment-related caries significantly decreased in children who had more opportunities to be visited by PCPs, compared to children who were only visited by dentists [33]. The results of the present study also indicated that only increasing number of dentists is not a feasible strategy for prevention and treatment of dental diseases, especially in younger children, unless an integrated system for provision of oral health services and fundamental executive programs are taken into account.

### Strengths and limitations

The most significant strength of the current study was its nationwide design and participation of a representative sample of young children from all provinces across country. The participants had an extensive variation of socioeconomic characteristics and dental caries experiences which could increase generalizability of the results to other developing countries with similar SES. Some limitations were also considered in this study. First, the cross-sectional design of study could not report causal associations. Moreover, we only examined children aged 6 and 12 years old. Finally, some effective factors were not controlled in the study including dietary habits, knowledge and attitude about oral health, self-perceived status of dental health, and oral-health related quality of life.

## Conclusion

In conclusion, this national survey found that economic factors had greater influence on maintenance and promotion of oral health in comparison with social determinants. However, socio-demographic factors and dental health behaviors were also effective in oral health maintenance. Therefore, oral health-based supportive policies _in terms of children national comprehensive programs; such as special oral hygiene packages and dental health educational programs particularly for those who encounter economic disparities_ could increase oral health literacy, improve dental health behaviors, and eventually maintain oral health habits; which might be of great concern to dental health policy makers.

## Data Availability

The datasets analyzed in the current study, derived from National Oral Health survey, are available on reasonable request from the corresponding author. Also economic datasets used are publically available through the website noted below https://www.amar.org.ir/english/Iran-Statistical-Yearbook.

## Abbreviations

CPI: Consumer price index
DALY: Disability adjusted life year
dmft/DMFT: decayed, missing and filled teeth in primary/permanent dentitions
GBD: Global burden of disease
GDP: Gross domestic product
PCPs: Primary care providers
SE: Standard error
SES: Socio-economic status
WHO: World Health Organization
